# Induction of mast cell accumulation, histamine release and skin edema by N49 phospholipase A_2_

**DOI:** 10.1186/1471-2172-10-21

**Published:** 2009-04-28

**Authors:** Ji-Fu Wei, Xiao-Long Wei, Ya-Zhen Mo, Shao-Heng He

**Affiliations:** 1 Clinical Experiment Center, the First Affiliated Hospital of Nanjing Medical University, Nanjing, 210029, PR China; 2 Allergy and Inflammation Research Institute, the Shantou University Medical College, Shantou, Guangdong, 515041, PR China

## Abstract

**Background:**

It has been recognized that phospholipase A_2 _(PLA_2_) is a crucial component of snake venom, which contributes greatly to snake venom induced inflammation in man. However, the mechanisms through which N49 PLA_2 _provoke inflammation remain unclear. Recently, a N49 PLA_2_, TM-N49 from *Protobothrops mucrosquamatus *crude venom was characterized in our laboratory. Since the purification procedure developed is able to supply us with relatively large quantity of highly purified TM-N49, we investigated the ability of TM-N49 in induction of inflammation.

**Results:**

The results showed that TM-N49 provoked a dose dependent increase in microvascular leakage in the skin of rats. The potency of TM-N49 in induction of skin edema appeared similar potency of bradykinin and histamine. Pretreatment of rats with compound 48/80 diminished TM-N49 induced skin reaction and reduced mast cell numbers in rats. Ginkgolide B and cyproheptadine, but not terfenadine and quinacrine, inhibited TM-N49 elicited microvascular leakage when they were co-injected with the stimulus to rat skin. Moreover, TM-N49 was found to induce histamine release from human colon, lung and tonsil mast cells, and both metabolic inhibitors and pertussis toxin were capable of inhibiting TM-N49 elicited histamine release. TM-N49 induced mast cell accumulation in the peritoneum of mice, which was inhibited by co-injection of ginkgolide B, cyproheptadine and terfenadine. Intravenous injection of monoclonal antibodies against CD18, ICAM-1 and CD11a also blocked TM-N49 induced mast cell accumulation.

**Conclusion:**

TM-N49 is a potent stimulus for skin edema, mast cell activation and accumulation.

## Background

Snake venoms are chemically complex mixtures of pharmacologically active proteins or peptides, which serve not only as a source of digestive enzymes, but also play an important role in immobilizing the prey and acting as offensive weapons. They can target multiple tissues, causing simultaneous damage of multiple physiological systems. One of the components which contribute significantly to the lethality of snake venoms is phospholipase A_2 _(PLA_2_) (EC 3.1.1.4) [1]. PLA_2 _constitutes a family of structurally related proteins hydrolyze phospholipids at the sn-2 position in a calcium-dependent manner, releasing fatty acids and lysophospholipids [2]. Snake venom PLA_2_s are low-molecular weight (13,000–14,000 Da), secretory phospholipases containing seven disulfide bonds. Based on their amino acid sequence and disulfide bond pattern, snake venom PLA_2_s are classified into group I PLA_2 _(from Elapidae/Hydrophidae) or group II PLA_2 _(from Crotalidae/Viperidae) [3]. Usually, the group II PLA_2_s are further subdivided into two major subgroups: the Asp-49 PLA_2_s (D49 PLA_2_s), which have an aspartic acid at position 49 and high catalytic activity towards artificial phospholipid substrates; and Lys-49 PLA_2_s (K49 PLA_2_s), which have a lysine substitutes at position 49 and very low or no hydrolytic activity towards artificial phospholipid substrates [4,5]. Recently, a unique subgroup of snake venom group II PLA_2_, named N49 PLA_2 _subgroup was identified from several Asiatic snake venoms [6-8]. The N49 PLA_2 _was found to differ from the other subgroups in its structure and biological activities.

Besides the digestive function, snake PLA_2_s exhibit severalother pharmacological properties including antiplatelet [9,10], anticoagulant [11], hemolytic [9], neurotoxic (presynaptic) [12], myotoxic [13-15]. They have also been employed widely as pharmacological tools to investigate the roles of these enzymes in diverse models of experimental inflammatory processes such as edema, inflammatory cell infiltration and mast cells activation [15-20]. Mast cells are primarily located in mucosal and perivascular areas of various tissues, which play an important role in body defense processes. Recent studies found that mast cells can be activated by snake venom and release carboxypeptidase A and possibly other proteases, which can degrade venom components [21,22]. Our former study also showed that atrahagin, a metalloprotienase purified from *Naja atra *snake, could potently activate human colon, lung and tonsil mast cells to release histamine [23]. Several snake venom PLA_2_s were reported to be able to activate the rat mast cells, to induce microvascular leakage and inflammatory cell accumulation at the sites of inflammation [15-20]. However, little is known of the action of N49 PLA_2_s on human mast cells, and the mechanisms through which N49 PLA_2 _induces microvascular leakage and inflammatory cell accumulation still remain obscure. Therefore, we investigated the mechanisms of TM-N49 [6] in induction of microvascular leakage and mast cell accumulation and activation in the present study.

## Results

### Purification and characterization of TM-N49

Approximately 15 mg of TM-N49 was obtained from 1.5 g *Protobothrops mucrosquamatus *crude venom following the procedures described above. The purity of the PLA_2 _was at least 98% as assessed by SDS-PAGE, HPLC and mass spectrometry analysis.

### Induction of microvascular leakage by TM-N49

**Figure 1:**
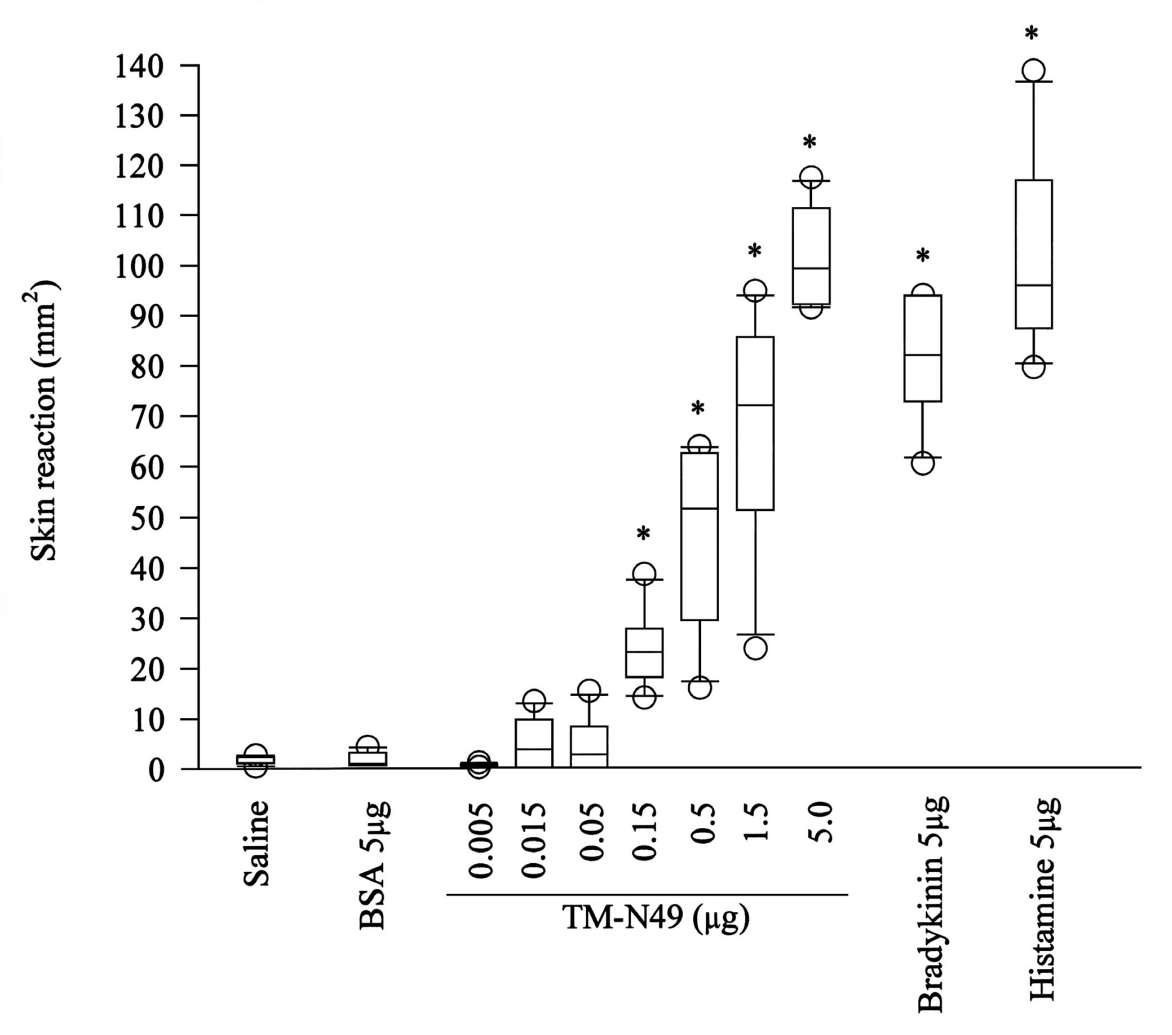
**Effect of TM-N49 on rat dermal microvascular leakage**. Various doses of TM-N49 were injected into the skin of rat for 20 min. Also shown are the responses to BSA, bradykinin and histamine alone at a dose of 5 μg and normal saline control. Skin reaction represented the area of Evan's blue extravasation. Data are displayed as a boxplot, which indicates the median, interquartile range, the largest and smallest values other than outliers (O) (defined as those which are more than 1.5 box lengths from the median) for 6 animals in each group. * *P *< 0.05 compared with the response to the diluent only control animals.

**Figure 2:**
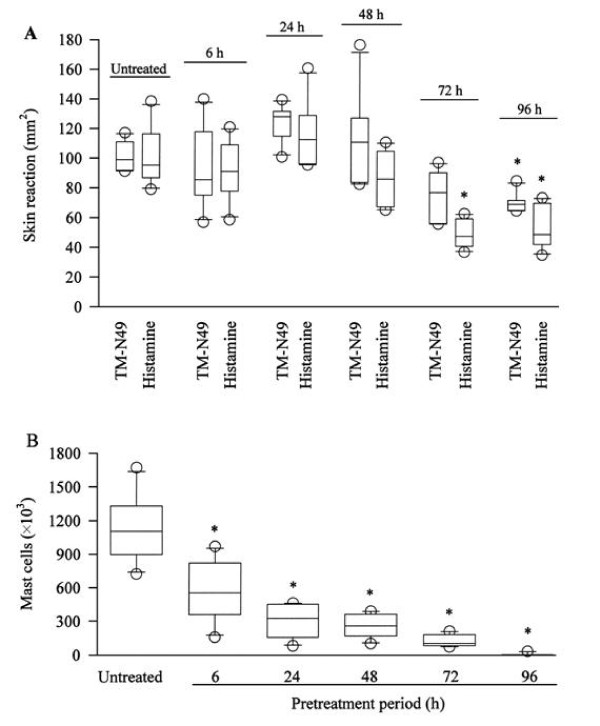
**Influence of compound 48/80 on rat dermal microvascular leakage induced by TM-N49 and histamine (A), and on mast cell numbers in the peritoneum of rats (B)**. Rats were intra-peritoneally injected with compound 48/80 at a dose of 0.6 mg·kg^-1 ^for 6 h, twice a day for 3 days, and doubled dose in day 4 before TM-N49 (5 μg) or histamine (5 μg) being administrated for 20 min. Skin reaction represented the area of Evan's blue extravasation. Data are displayed as a boxplot, which indicates the median, interquartile range, the largest and smallest values other than outliers (O) (defined as those which are more than 1.5 box lengths from the median) for 6 animals in each group. In (**A**), * *P *< 0.05 compared with the corresponding response to untreated animals and in (**B**), * *P *< 0.05 compared with the response to untreated animals.

TM-N49 at doses of 0.15–5.0 μg provoked a dose dependent increase in microvascular leakage in the skin of rats at 20 min following injection. As little as 0.15 μg was able to stimulate significant skin edema after injection indicating that TM-N49 is a potent stimulus. The potency of TM-N49 in induction of skin edema is similar to that of bradykinin and histamine on the weight basis (all at 5 μg) (Figure 1). Pretreatment of rats with compound 48/80 for a period longer than 72 h clearly diminished the skin responsiveness of the rats to TM-N49 and histamine (Figure 2A), and dramatically reduced mast cell numbers in the peritoneal lavage fluid of these rats (Figure 2B).

### Influence of anti-inflammatory compounds on microvascular leakage

**Figure 3:**
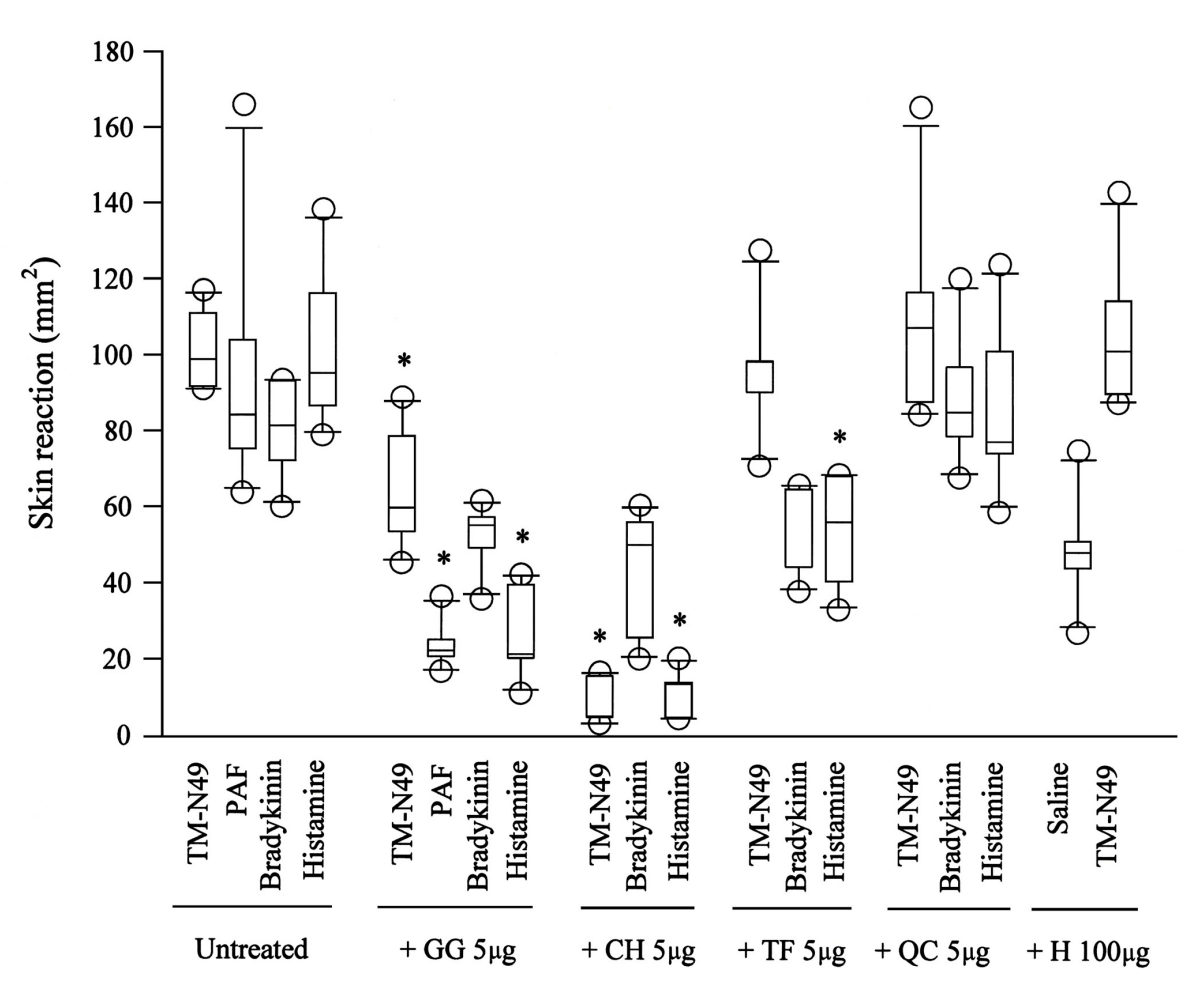
**Influence of anti-inflammatory drugs on rat dermal microvascular leakage induced by TM-N49 (5 μg), bradykinin (5 μg), PAF (5 μg) and histamine (5 μg)**. Ginkgolide (GG, 5 μg), cyproheptadine (CH, 5 μg), terfenadine (TF, 5 μg) or quinacrine (QC, 5 μg) were co-injected with TM-N49, bradykinin or histamine, respectively for 20 min, whereas PAF was only co-injected with GG. Skin reaction represented the area of Evan's blue extravasation. Data are displayed as a boxplot, which indicates the median, interquartile range, the largest and smallest values other than outliers (O) (defined as those which are more than 1.5 box lengths from the median) for 6 animals in each group. * *P *< 0.05 compared with the response to the corresponding uninhibited control animals.

Ginkgolide B at a dose of 5 μg inhibited 73.5%, 77.5% and 40% microvascular leakage induced by PAF, histamine and TM-N49, respectively when it was co-injected with these stimuli. Cyproheptadine at a dose of 5 μg also inhibited 85.6% and 94.7% histamine and TM-N49 elicited microvascular leakage. Terfenadine and quinacrine at the dose of 5 μg had little effect on TM-N49 provoked microvascular leakage in rat skin (Figure 3). All anti-inflammatory compounds tested by themselves had no significant effect on the microvascular leakage in rat skin (data not shown).

### Induction of histamine release from mast cells by TM-N49

**Figure 4:**
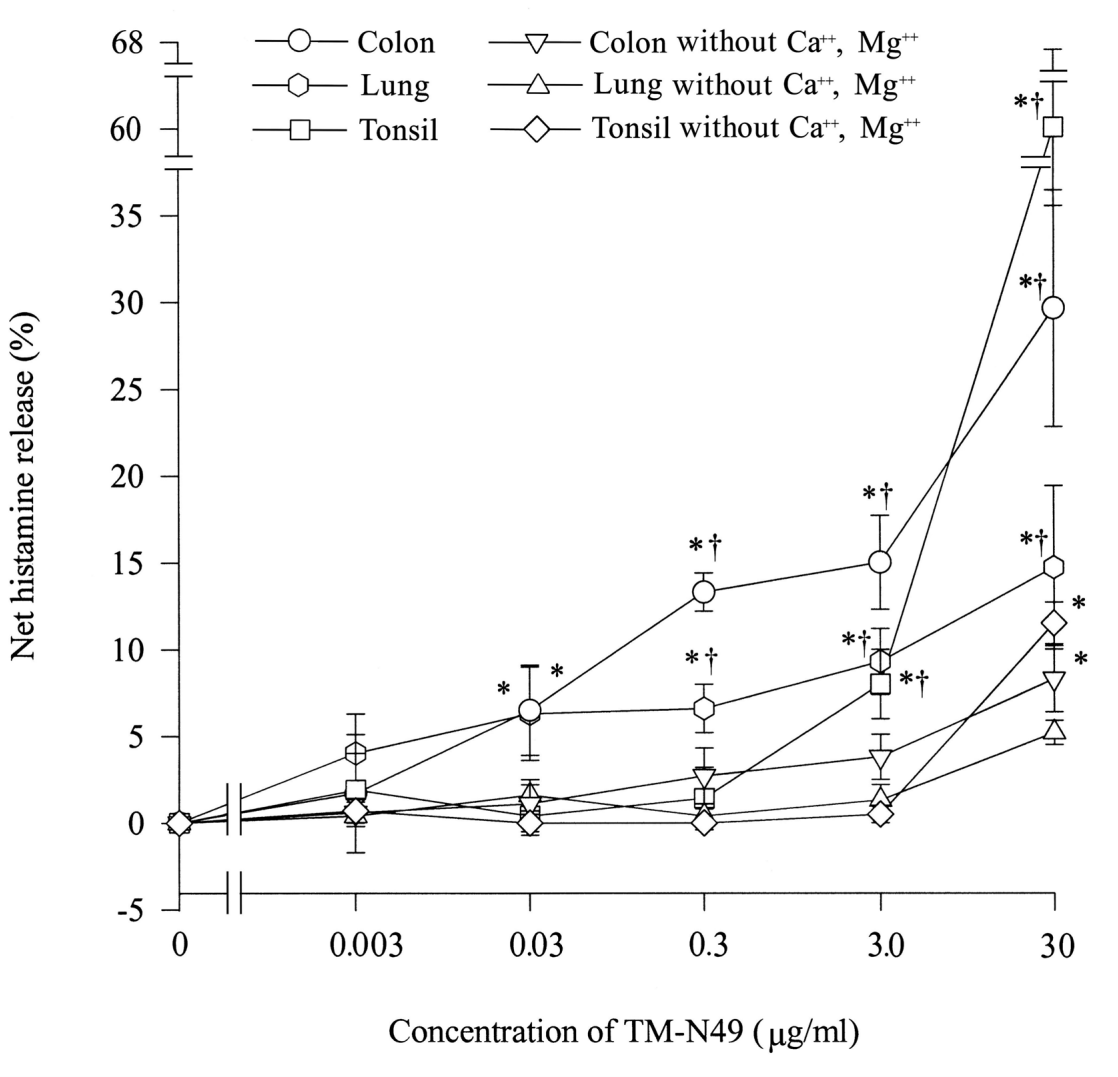
**Effects of TM-N49 on histamine release from colon, lung and tonsil mast cells in the presence or absence of exogenous calcium and magnesium**. The values shown are mean ± SE for four to five separate experiments. Various concentrations of TM-N49 were incubated with cells for 15 min before termination of the reactions. * *P *< 0.05 compared with the response to the corresponding buffer alone group, ^†^*P *< 0.05 compared with the response to the corresponding group in the absence of calcium and magnesium.

A dose dependent release of histamine from colon, lung and tonsil mast cells was observed when various concentrations of TM-N49 were incubated with cells for 15 min. As low as 0.03 μg/ml of TM-N49 was able to stimulate significant histamine release from human colon and lung mast cells, but to stimulate a similar level of histamine release from tonsil mast cells a minimum of 3.0 μg/ml of TM-N49 was required. While TM-N49 at a concentration of 30 μg/ml was able to provoke approximately 30%, 15% and 62% net histamine release, anti-IgE antibody at a concentration of 10 μg/ml stimulated approximately 17%, 17% and 11% net histamine release from colon, lung and tonsil mast cells, respectively in the parallel experiments. At a concentration of 30 μg/ml, TM-N49 induced also significant histamine release from colon and tonsil mast cells when calcium and magnesium were absent in the challenge buffer (Figure 4).

### Time course for TM-N49 induced histamine release

**Figure 5:**
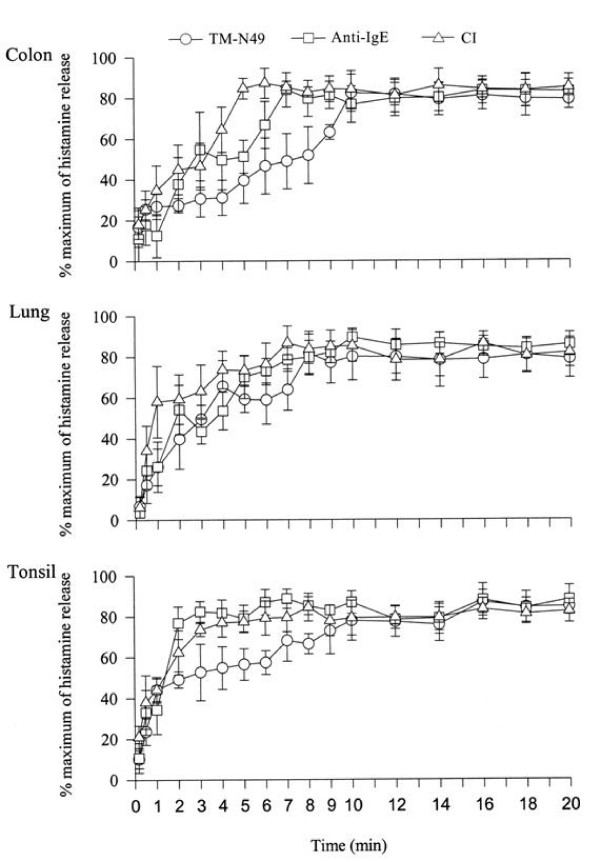
**Time course for histamine release from colon, lung and tonsil mast cells induced by TM-N49 (30 μg/ml), anti-IgE antibody (10 μg/ml) and calcium ionophore (1 μg/ml)**. The values shown are mean ± SE of the percentage of maximum histamine release, which equals (actual net histamine release/maximum net histamine release induced by the testing compound) × 100%, for four to five separate experiments.

Immediately after adding 30 μg/ml of TM-N49 to cells, histamine release from colon, lung and tonsil mast cells occurred. Approximately 26.8%, 26.1% and 44.1% of the maximum of histamine release were observed following incubation of TM-N49 with colon, lung and tonsil cells, respectively for 1 min. At the same time point, anti-IgE antibody at 10 μg/ml induced 12.5%, 26.2% and 34.3% of the maximum of histamine release, and calcium ionophore at a concentration of 1 μg/ml provoked 34.7%, 58.1% and 44.1% of the maximum of histamine release from colon, lung and tonsil mast cells, respectively (Figure 5).

The peak of histamine released from colon, tonsil and lung mast cells in response to TM-N49 occurred at 10, 10 and 8 min following TM-N49 being added to cells, whereas in the parallel experiments, the peak histamine release induced by anti-IgE antibody occurred at 7, 10 and 7 min for colon, lung and tonsil mast cells, respectively. Similarly, the peak histamine release provoked by calcium ionophore appeared at 6, 7 and 8 min for colon, lung and tonsil mast cells, respectively. The maximum release of histamine was then sustained at least to 20 min (Figure 5).

### Effects of metabolic inhibitors and pertussis toxin on histamine release

When cells were pretreated with metabolic inhibitors for 40 min 3 μg/ml of TM-N49 induced histamine release from colon, lung and tonsil mast cells was completely abolished. However, metabolic inhibitors exhibited little effect on histamine release from tonsil mast cells when 30 μg/ml of TM-N49 was added to cells. Similarly, metabolic inhibitors dramatically inhibited anti-IgE antibody provoked histamine release from colon, lung and tonsil mast cells, but had relatively less efficient inhibition of calcium ionophore elicited histamine release from mast cells (Table 1). Pretreatment of cells with pertussis toxin for 4 h reduced dramatically their responses to TM-N49 and anti-IgE antibody, but had no significant effect on their ability to release histamine in response to calcium ionophore (Table 2).

**Table 1:** The effect of metabolic inhibitors on TM-N49 induced histamine release from human mast cells

Compound (μg/ml)		Net histamine release (%)					
		Without metabolic inhibitors			With metabolic inhibitors		
		Lung	Colon	Tonsil	Lung	Colon	Tonsil
TM-N49	3.0	9.3 ± 1.9	13.5 ± 3.0	8.0 ± 2.0	0.4 ± 0.4*	0.4 ± 0.7*	0.8 ± 0.6*
	30	14.7 ± 4.7	29.6 ± 6.8	62.0 ± 8.5	1.6 ± 0.9*	0.4 ± 1.4*	49.3 ± 7.5
Anti-IgE	10	17.3 ± 5.4	17.4 ± 5.1	11.1 ± 3.4	0.7 ± 1.7*	0.1 ± 0.8*	2.2 ± 1.5*
CI	0.5	24.4 ± 6.3	38.8 ± 4.6	37 ± 9.3	5.3 ± 5.5*	2.1 ± 0.7*	6.4 ± 2.9*

The values shown are mean ± SE for four to five separate experiments performed in duplicate. Cells were incubated with metabolic inhibitors 2-deoxy-D-glucose (10 mM) and antimycin A (1 μM) for 40 min at 37°C before challenging with stimulus. * *P *< 0.05 compared with the corresponding uninhibited controls.

**Table 2:** The effect of pertussis toxin on TM-N49 induced histamine release from human mast cells

Compound (μg/ml)		Net histamine release (%)					
		Without pertussis toxin			With pertussis toxin		
		Lung	Colon	Tonsil	Lung	Colon	Tonsil
TM-N49	3.0	9.3 ± 1.9	13.5 ± 3.0	8.0 ± 2.0	0.8 ± 1.8*	0.1 ± 2.2*	1.5 ± 1.3*
	30	14.7 ± 4.7	29.6 ± 6.8	62.0 ± 8.5	4.0 ± 0.6*	3.9 ± 3.9*	31.9 ± 9.2*
Anti-IgE	10	17.3 ± 5.4	17.4 ± 5.1	11.1 ± 3.4	2.1 ± 1.1*	0.9 ± 1.2*	3.0 ± 0.3*
CI	0.5	24.4 ± 6.3	38.8 ± 4.6	37 ± 9.3	19.6 ± 4.8	30.4 ± 2.8	20.4 ± 4.4

The values shown are mean ± SE for four to five separate experiments performed in duplicate. Cells were incubated with1.0 μg/ml pertussis toxin for 4 h at 37°C before challenging with stimulus. * *P *< 0.05 compared with the corresponding uninhibited controls.

### Induction of mast cell accumulation by TM-N49

**Figure 6:**
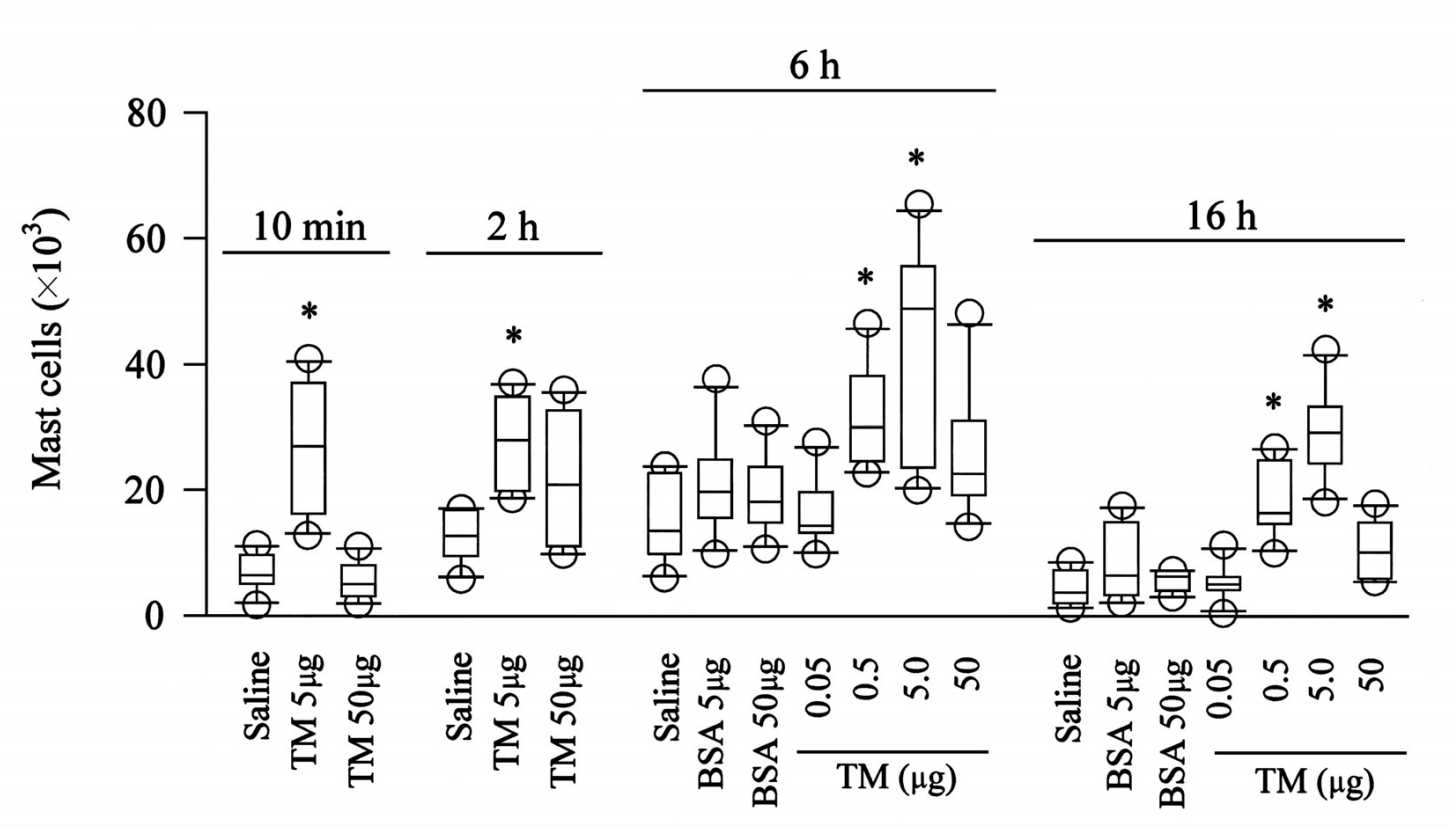
**Effect of TM-N49 (TM) on mast cell numbers in mouse peritoneum**. Various doses of TM-N49 were injected into the peritoneum of mice for 10 min, 2 h, 6 h or 16 h. Also shown are the responses to BSA and normal saline control. Data are displayed as a boxplot, which indicates the median, interquartile range, the largest and smallest values other than outliers (O) (defined as those which are more than 1.5 box lengths from the median) for 6 animals in each group. * *P *< 0.05 compared with the response to the corresponding diluent only control animals.

As early as 10 min following injection, 5 μg of TM-N49 was able to induce significant mast cell accumulation in the peritoneum of mice. The mast cell accumulation induced by TM-N49 appeared to at least maintain for 16 h. As little as 0.5 μg of TM-N49 was able to potently induce mast cell infiltration in the peritoneum of mice at 6 h and 16 h following injection (Figure 6). However, relative mast cell number in mouse peritoneum was not significantly increased except for 5 μg of TM-N49 injected for 2 h. At 50 μg, TM-N49 markedly reduced relative number of mast cells at all the time points examined following injection (Table 3).

**Table 3:** Effect of TM-N49 on relative mast cell numbers in mouse peritoneum following injection.

TM-N49 (μg)	Percentage of mast cells in total nucleated cells			
	
	10 min	2 h	6 h	16 h
Saline	0.49(0.40–0.96)	0.45(0.31–1.30)	0.56(0.23–0.76)	0.85(0.29–1.88)
50	0.16(0.06–0.35)*	0.24(0.08–0.78)*	0.36(0.21–0.90)	0.05(0.03–0.11)*
5.0	0.76 (0.44–1.11)	1.05(0.60–1.77)*	0.67(0.29–1.76)	0.51 (0.22–1.36)
0.5	n.d	n.d	0.61(0.39–1.00)	0.63 (0.27–1.10)
0.05	n.d	n.d	0.90(0.39–2.67)	1.10 (0.47–1.31)

Various doses of TM-N49 were injected into the peritoneum of mice for 10 min, 2 h, 6 h or 16 h. Also shown are the responses to normal saline control. Relative number of mast cells was defined as the percentage of mast cell in total nucleated cells. * *P *< 0.05 compared with the response to saline alone.

### Effects of anti-inflammatory compounds and blocking antibodies on mast cell accumulation

When co-injected, ginkgolide B, cyproheptadine and terfenadine inhibited 85.6%, 80% and 100% TM-N49 induced mast cell accumulation in the peritoneum of mice, respectively. However, quinacrine did not significantly alter the extent of TM-N49 induced mast cell accumulation. At the dose tested, ginkgolide B, cyproheptadine, terfenadine and quinacrine by themselves failed to induce mast cell accumulation in the peritoneum of mice (Table 4).

**Table 4:** The influence of anti-inflammatory compounds on TM-N49 (5 μg) induced mast cell accumulation in mouse peritoneum

Compound injected		Number of mast cells (× 10^3^)
Saline		14.6 (6.1 – 23)
TM-N49		49.3 (20.3 – 65.9)
Ginkgolide B	5 mg·kg^-1^	26.2 (9.1 – 41)
Ginkgolide B	5 mg·kg^-1 ^+ TM-N49	19.6 (10 – 26)*
Cyproheptadine	2 mg·kg^-1^	6.2 (1.5 – 17.2)
Cyproheptadine	2 mg·kg^-1 ^+ TM-N49	21.9 (10.1 – 28.7)*
Terfenadine	2 mg·kg^-1^	11.0 (5.8 – 20)
Terfenadine	2 mg·kg^-1 ^+ TM-N49	9.5 (4.0 – 13.2)*
Quinacrine	10 mg·kg^-1^	11.5 (5.9 – 18.1)
Quinacrine	10 mg·kg^-1 ^+ TM-N49	59.7 (40.5 – 73.3)

The values shown are medians (range) for six separate experiments. Compounds were injected into the mouse peritoneum for 6 h before peritoneal lavage fluid being collected. * *P *< 0.05 compared with the response to TM-N49 alone.

Intravenous injection of monoclonal antibodies against CD18, ICAM-1 and CD11a 30 min prior to intra-peritoneal injection of the PLA2 blocked TM-N49 induced mast cell accumulation by 70% and 99%, respectively. Monoclonal antibodies against L-selectin failed to diminish TM-N49 induced mast cell accumulation in the peritoneum of mice. Normal rat and hamster IgG isotype controls tested had little effect on TM-N49 induced mast cell accumulation (Table 5).

**Table 5:** The influence of blocking antibodies (Ab) against cell adhesion molecules on TM-N49 (5.0 μg) induced mast cell accumulation in mouse peritoneum

Compound injected		Number of mast cells (× 10^3^)
Saline		14.6 (6.1 – 23)
TM-N49		49.3 (20.3 – 65.9)
L-selectin Ab	+ TM-N49	38.0 (19.1 – 68.8)
LFA-1 Ab	+ TM-N49	33.7 (18.9 – 55.2) *
CD18 Ab	+ TM-N49	25.1 (10.4 – 39.8)*
ICAM-1 Ab	+ TM-N49	14.8 (9.6 – 32.0)*
Hamster IgG1	+ TM-N49	39.8 (17.0 – 63.1)
Rat IgG2a	+ TM-N49	43.3 (19.3 – 58.0)

The values shown are medians (range) for six separate experiments. Monoclonal antibodies (all at a dose of 1 mg·kg^-1^) against the adhesion molecules L-selectin, LFA-1, CD18 and ICAM-1 were intravenously injected, respectively for 30 min before intra-peritoneal injection of 5 μg of TM-N49 for 6 h. * *P *< 0.05 compared with the response to TM-N49 alone.

## Discussion

As a novel subgroup of snake venom group II PLA_2_, the proinflammatory activities of N49 PLA_2 _have not yet been studied. Therefore, this is the first study to investigate the potential actions of N49 PLA_2 _in inflammation. TM-N49 was found to be a potent stimulus of microvascular leakage in the skin of rats. The potency of this unique, inactive PLA_2 _in induction of microvascular leakage is similar to the potency of bradykinin and histamine on a molar basis. Therefore it is not difficult to anticipate that TM-N49 by itself should be able to induce severe inflammatory reactions upon snake bite.

Since histamine/5-HT antagonist cyproheptadine abolished the TM-N49-induced microvascular leakage, but selective histamine H_1 _receptor antagonist terfenadine did not, the action of TM-N49 on skin edema seems to be histamine independent. It is likely that 5-HT and other mast cell products, such as PAF, serine proteases, leukotrienes, prostaglandins are involved in the process [24]. Indeed, it was observed that ginkgolide B, a PAF receptor antagonist inhibited microvascular leakage induced by TM-N49, suggesting that PAF was involved in the microvascular leakage process. Mast cell mediated edema formation in response to different snake venom PLA_2 _has been reported previously. Thus, PLA_2 _from *Vipera russeli*, *Naja mocambique *and honey bee produced a rapid but transient oedematous response in rat paw [25]. Bothropstoxin-I and bothropstoxin-II, two K49 PLA_2_s isolated from *Bothrops jararacussu *snake venom caused dose-dependent rat paw and skin oedema formation [17]. PLA_2_s from *Crotalus durissus cascavella*, *Crotalus durissus collilineatus *and *Crotalus durissus terrificus *venoms increased the vascular permeability in the rat skin [26]. However, the observed reduction of mast cell numbers by injection of compound 48/80 hardly affects the inflammatory potential of TM-N49: even a 10-fold reduction in detectable mast cells does not result in a significant reduction of extravasation. This implied that mast cell activation may not be the relevant mechanism for TM-N49 induced skin edema. It is difficult to exclude the possibility that TM-N49 bound to a receptor of PLA_2 _and caused microvascular leakage, though the latter has not yet been identified. Recently, it was found that snake venom catalytically inactive PLA_2 _homologue could binds to vascular endothelial growth factor receptor-2 via a C-terminal loop region, exhibiting mast cell unrelated mechanism for the increased the vascular permeability [27].

In order to study the activation of mast cells by TM-N49, mast cell challenge experiments were performed. TM-N49 can activate human colon, lung and tonsil mast cells in a dose dependent manner. It was discovered further that TM-N49 induced mast cell degranulation is an energy consuming process and involved a G protein coupled receptor in mast cells [28] as both metabolic inhibitors and pertussis toxin were capable of inhibiting TM-N49 elicited histamine release from mast cells. It was reported previously that PLA_2_s were able to directly activate rat mast cells *in vitro *[17,20,29]. The action of D49 PLA_2_s on rat mast cells was dependent on their catalytic activity [16,30], whereas the influence of K49 PLA_2_s, which possess negligible catalytic activity, on rat mast cells was through a heparin-sensitive mechanism [17]. Removal of Ca^2+ ^and Mg^2+ ^from challenge buffer greatly reduced the cell response to the stimulation of TM-N49 indicated that exogenous calcium and magnesium ions were crucial for mast cell degranulation induced by TM-N49. The increased serotonin level in mouse peritoneal lavage fluid supplied further evidence on TM-N49 causing mast cell degranulation in rats as mast cells are major source (if not the only source) of serotonin in mouse peritoneum. The most relevant mast cell population in regard to snake bites would be skin mast cells which differ in phenotype and activating stimuli from other mast cell populations. From those populations investigated, tonsil mast cells most closely resemble skin mast cells [31]. Interestingly, as shown in figure 4, tonsil mast cells only responded to rather high concentrations of TM-N49, and the overall release (~7%) is very little compared to other venom components (e.g. atrahagin, induces 26% histamine release) [23]. TM-N49 at 30 μg appears to be toxic, as the observed release is later shown to be Ca^2+^-independent. This, again, indicates that mast cell activation may not be the relevant mechanism for TM-N49 induced inflammation.

With a mouse peritoneal model, it was found for the first time that snake venom PLA_2 _could induce mast cell accumulation even at 10 min following injection. This appeared not a mast cell-specific effect since the relative number of mast cells in total nucleated cells was not significantly altered. The action of TM-N49 in induction of mast cell accumulation could be inhibited by co-injection of TM-N49 with ginkgolide B, cyproheptadine and terfenadine, suggesting that histamine, 5-HT and PAF may play a role in mast cell accumulation induced by TM-N49. Antibodies specific for CD18, CD11a and ICAM-1 blocked TM-N49 induced mast cell accumulation, while L-selectin specific antibody failed to do so. These observations suggested that LFA-1 (CD11a/CD18) and ICAM-1, but not L-selectin were involved in the TM-N49 induced mast cell migration process. Involvement of CD11a/CD18 and ICAM-1 in mast cell migration was reported previously with Saban et al. [32,33]. Since mast cells and endothelial cells express both LFA-1 and ICAM-1 [32,33], we believe that CD11/CD18 and ICAM-1 are important adhesion molecules for TM-N49 induced mast cell migration. The increase in mast cell numbers could be regarded as a beneficial host response since mast cells were shown to be able to detoxify venom components [21].

## Conclusion

A novel snake venom group II PLA_2_, TM-N49 was able to induce a dramatic increase in microvascular permeability in the skin of rats. The action of TM-N49 appeared to be unrelated to the activation of mast cells. Indeed, it was confirmed that TM-N49 was able to stimulate mast cell degranulation and accumulation. The ability of TM-N49 in induction of microvascular leakage, mast cell degranulation and mast cell infiltration implicates that it is a potent proinflammatory factor in snake venom.

## Methods

### Reagents

*Protobothrops mucrosquamatus *crude venom was obtained from the stock of the Kunming Institute of Zoology, the Chinese Academy of Sciences. SP-sephadex C-25, heparin sepharose (FF) and superdex 75 were from LKB Pharmacia (Uppsala, Sweden). The following compounds were purchased from Sigma (St. Louis, USA): egg phosphatidyl choline, Triton X-100, trifluoroacetic acid, honey bee venom phospholipase A2, platelet activating factor (PAF), cyproheptadine, ginkgolide B, heparin, collagenase (type I), hyaluronidase (type I), soybean trypsin inhibitor (SBTI), bovine serum albumin (BSA, fraction V), penicillin and streptomycin, calcium ionophore A23187, antimycin A, 2-deoxy-D-glucose, pertussis toxin. Quinacrine was from Calbiochem (San Diego, CA, USA). Reagents for sodium dodecyl- sulphate-polyacrylamine gel electrophoresis (SDS-PAGE) were from Bio-Rad Laboratories Inc (Hercules, USA). Coomassie Plus™ assay kit was from Pierce Chemical Co (Rockford, IL, USA). PolyATract system 1000 kit and Reverse transcription system kit were from Promega Biotech (Madison, WI, USA). Goat anti-human IgE (inactivated) was from Serotec (Kidlington, Oxford, UK). Fetal calf serum (FCS) and minimum essential medium (MEM) containing 25 mM *N*-2-hydroxylethylpiperazine-*N*'-2 -ethane sulphonic acid (HEPES) were from Gibco (Paisley, Renfrewshire, UK). Rat monoclonal antibodies, anti-mouse CD 11a [lymphocyte function-associated antigen 1(LFA-1) α chain], clone M17/4; anti-mouse CD 62L (L-selectin), clone MEL-14; anti-mouse CD18 (integrin β_2 _chain), clone M18/2; rat IgG2a isotype standard, clone R35-95; hamster anti-mouse CD54 [intercellular adhesion molecule 1 (ICAM-1)] monoclonal antibody, clone 3E2; hamster IgG1 isotype standard, clone A19-3 were from BD Biosciences Pharmigen (CA, USA). Hepes and all other chemicals were of analytical grade. BALB/c mice (20–25 g) and Wistar rat (180–220 g) were bred and reared under strict ethical conditions according to international recommendation.

### Purification of TM-N49

TM-N49 was isolated from *Protobothrops mucrosquamatus *crude venom following the procedures described previously [6]. Briefly, the lyophilized venom (1.5 g) was dissolved in 20 ml of 50 mM sodium phosphate buffer (pH 5.8) and loaded on a SP-sephadex C-25 column equilibrated with the same buffer. The absorbed proteins were eluted with a linear gradient of NaCl (0–0.8 M) in 50 mM sodium phosphate (pH 5.8). The fractions in peak 9 were collected, lyophilized, and then dissolved in a running buffer containing 25 mM sodium phosphate (pH 5.8) and 0.15 M NaCl. Superdex 75 column was used to further isolate TM-N49 and the fractions 50–56 in the main protein peak was collected and loaded on a heparin-sepharose (FF) column equilibrated with a 25 mM sodium phosphate buffer (pH 5.8). The absorbed proteins were eluted with a linear gradient of NaCl (0–0.8 M) in 25 mM sodium phosphate (pH 5.8). The fractions 40–47 in the main protein peak eluted from heparin agarose were collected, lyophilized, dissolved in 0.1% trifluoroacetic acid (v/v) and loaded on a reverse-phase C_18 _high performance liquid chromatography colomn (Symmetry 300™, 5 μM; Waters Corporation, Milford, Massachusetts, USA). The elution was performed with 0.1% trifluoroacetic acid and a gradient of 15–75% buffer B (containing 100% acetonitrile (v/v) and 0.1% trifluoroacetic acid) over 60 min at a flow rate of 1 ml/min. The fractions in main protein peak representing the purified TM-N49 were pooled, lyophilized and analyzed with mass spectrometer and stored at -20°C until use. Protein concentration was determined by a modified Bradford method [34] using a Coomassie Plus™ assay kit with BSA as standard. The PLA_2 _activity was routinely assayed by a titration method using egg yolk as substrate according to Ishimaru et al. [35], and by a colorimetric assay using L-phosphatidylcholine as substrate according to de Ajaujo and Radvanyi [36]. Honey bee PLA_2 _was employed as positive control.

### Induction of microvascular leakage

Microvascular leakage experiments were performed mainly following the procedures described previously by He et al. [37]. Briefly, Wistar rats (180–220 g) were anaesthetized by intra-peritoneal injection of sodium pentobarbitone at a dose of 21 mg·kg^-1^. After shaving the back, 1% (w/v) Evan's blue dye in normal saline was injected intravenously into the tail vein, and then various doses of TM-N49, bradykinin, histamine and PAF all at 5 μg, BSA at 5 μg and 50 μg or normal saline were intradermally injected, randomized sites placed 2–3 cm apart, 50 μl per injection point. Animals were kept warm, and at 20 min following the final injection, were killed by inhalation of carbon dioxide and the skin removed. Two perpendicular diameters were recorded for the blue area on the inside of the skin, and multiplied to give a measure of the relative size of the area of cutaneous edema.

For compound 48/80 pretreatment experiments, groups of rats (including 6 h, day 1, day 2, day 3, day 4 groups and their corresponding control groups) were treated with compound 48/80 to deplete mast cells, as described by Di Rosa et al. [38]. Briefly, compound 48/80 (0.1% solution in 0.9% sterile saline) was injected intravenously at a dose of 0.6 mg·kg^-1^, twice a day for 3 days, and doubled dose in day 4. At 5 h following the final injection, the test substances in the skin oedema experiments were administered as described above. The control groups received a normal saline injection. Both skins and peritoneal lavages of the testing rats were collected and analyzed.

To investigate potential mechanisms involved in TM-N49 induced microvascular leakage, several anti-inflammatory compounds including cyproheptadine (5 μg), a 5-HT_2_/5-HT_1C_serotonin receptor antagonist and H_1 _histamine receptor antagonist; terfenadine (5 μg), a selective H_1 _histamine receptor antagonist; ginkgolide B (5 μg), a PAF receptor antagonist [39] and quinacrine (5 μg), an inhibitor of PLA_2 _[40] were co-injected into the skin of rats with TM-N49 PLA_2 _(5 μg), bradykinin (5 μg), histamine (5 μg), or normal saline respectively for 20 min.

### Mast cell challenge and analysis of histamine release

Macroscopically normal lung and colon tissues were collected at bronchial or colon resection from patients with lung or colon cancer, respectively, and tonsil tissue was obtained at tonsillectomy. The procedure for mast cell dispersion was similar to that described previously [23,28]. Briefly, tissue was chopped finely with scissors into fragments of 0.5–2.0 mm^3^, and incubated with 1.5 mg/ml collagenase and 0.75 mg/ml hyaluronidase in MEM containing 2% FCS (1 g lung/10 ml buffer) for 70 min at 37°C. Dispersed cells were separated from undigested tissue by filtration through nylon gauze (pore size 100 μm diameter), and were maintained in MEM (containing 10% FCS, 200 U/ml penicillin, 200 μg/ml streptomycin) on a roller overnight at room temperature. Mast cell numbers were determined by light microscopy after staining with alcine blue staining solution, and represented 2.5 to 5.3%, 4.2 to 5.7%, and 1.2 to 3.1% of nucleated cells in lung, colon or tonsil suspensions, respectively.

Prior to challenge with stimulus the cells were washed with HBSS (pH 7.4) without added calcium or magnesium (500 g, 10 min, 25°C), and then resuspended in HBSS with 1.8 mM CaCl_2 _and 0.5 mM MgCl_2_. Aliquots of 100 μl containing 4–6 × 10^3 ^mast cells were added to a 50 μl aliquot of purified TM-N49, heparin, or control secretagogue in complete HBSS and incubated for 15 min at 37°C. The reaction was terminated by the addition of 150 μl ice cold HBSS and the tubes centrifuged immediately (500 g, 10 min, 4°C). All experiments were performed in duplicate. For the measurement of total histamine concentration the suspension in some tubes was boiled for 6 min. Supernatants were stored at -20°C until histamine concentrations were determined. Where added, TM-N49 at 30 μg/ml was preincubated with heparin on ice for 10 min before being added to cells.

For the experiments with pertussis toxin, cells were incubated with 0.1 or 1.0 μg/ml pertussis toxin for 4 h at 37°C, and then washed with HBSS before adding stimulus. Similarly, for the experiments with metabolic inhibitors, cells were incubated with 2-deoxy-D-glucose (10 mM) and antimycin A (1 μM) for 40 min at 37°C before challenged with stimulus.

A glass fibre-based, fluorometric assay was employed to determine histamine levels in supernatants, as previously described [23,28]. Histamine bound to a glass-fibre matrix (RefLab, Copenhagen, Denmark) was detected by addition of o-phthaldialdehyde (OPT) and the color change measured using a spectrophotofluorometer (Perkin-Elmer LS 2, Denmark). Histamine release was expressed as a percentage of total cellular histamine levels, and corrected for the spontaneous release measured in tubes in which cells had been incubated with the HBSS diluent alone. For the time course study, data were presented as percentage of maximum histamine release, which equals (actual net histamine release/maximum net histamine release induced by the testing compound) × 100%.

### Induction of mast cell accumulation

Various doses of TM-N49, BSA or normal saline were injected in 0.5 ml volumes into the peritoneum of male BALB/c mice, whose abdominal skin was swabbed with 70% ethanol, a group of 6 mice for each dose. This model was adapted from that described by Thomas et al. [41], which complied with the European Community guidelines for use of experimental animals. At 10 min, 2 h, 6 h or 16 h following injection, animals were sacrificed by inhalation of carbon dioxide, and their peritoneal lavages were collected following a standardized procedure with 5 ml normal saline using heparinised tubes. After centrifugation at 500 g for 10 min at 4°C, supernatants were collected and stored at -40°C until use, and cells were resuspended in 1 ml of MEM. The total cell numbers were determined by enumerating them with an Improved Neubauer haemocytometer after being stained with 0.1% trypan blue. For the differential cell counting, cytocentrifuge preparations were made, air dried and stained with modified Wright's stain. Differential cell counts were performed for a minimum of 500 cells. The results were expressed as absolute numbers of each cell type per mouse peritoneum.

For the experiments investigating mast cell migration mechanisms, groups of mice were pretreated intravenously (tail vein injection) with monoclonal antibodies against the adhesion molecules L-selectin, CD11a, CD18 and ICAM-1 (all at a dose of 1 mg·kg^-1^) [42-44], respectively for 30 min before intra-peritoneal injection of 5 μg TM-N49. Control animals received an equivalent dose of the corresponding normal rat or hamster IgG isotype control. At 6 h following injection, the mice were sacrificed and their peritoneal lavages were processed as described above.

To investigate potential mechanisms involved in TM-N49 induced inflammatory cell accumulation, several anti-inflammatory compounds including cyproheptadine (2 mg·kg^-1^) [17], terfenadine (3 mg·kg^-1^) [45,46], ginkgolide B (5 mg·kg^-1^) [39] and quinacrine (10 mg·kg^-1^) [40] were co-injected into the peritoneum of mice with TM-N49 (5 μg per mouse). Control animals received an injection of drug alone. At 6 h following injection, mice were sacrificed and their peritoneal lavages were processed as described above. Certain mice were pretreated with compound 48/80 for 4 days as described above before peritoneal injection of 5 μg of TM-N49.

### Statistical analysis

Statistical analyses were performed using SPSS software (version 12.0). For *in vitro *experiments, because data were normally distributed, they are shown as the mean ± SEM. for the number of experiments indicated. Where analysis of variance indicated significant differences between groups, for the preplanned comparisons of interest, paired Student's *t *test was applied. For *in vivo *experiments, since the data were not normally distributed, they were presented as the median and range, for the numbers of animals indicated. Where Kruskal-Wallis analysis indicated significant differences between groups, for the preplanned comparisons of interest the Mann-Whitney *U*-test was employed. For all analyses, *P *< 0.05 was taken as significant.

## Authors' contributions

JFW and SHH conceived and designed the study. JFW, XLW and YZM performed the experiments. JFW drafted the manuscript and SHH revised the manuscript. SHH supervised and coordinated the whole project. All authors have read and approved the final manuscript.
